# The first complete plastid genomes of Melastomataceae are highly structurally conserved

**DOI:** 10.7717/peerj.2715

**Published:** 2016-11-29

**Authors:** Marcelo Reginato, Kurt M. Neubig, Lucas C. Majure, Fabian A. Michelangeli

**Affiliations:** 1 Institute of Systematic Botany, The New York Botanical Garden, Bronx, New York, United States; 2 Department of Plant Biology, Southern Illinois University of Carbondale, Carbondale, Illinois, United States; 3 Department of Research, Conservation and Collections, Desert Botanical Garden, Phoenix, Arizona, United States

**Keywords:** Myrtales, Chloroplast, Melastomataceae, Plastome, Genome skimming, Phylogenomics, NGS

## Abstract

**Background:**

In the past three decades, several studies have predominantly relied on a small sample of the plastome to infer deep phylogenetic relationships in the species-rich Melastomataceae. Here, we report the first full plastid sequences of this family, compare general features of the sampled plastomes to other sequenced Myrtales, and survey the plastomes for highly informative regions for phylogenetics.

**Methods:**

Genome skimming was performed for 16 species spread across the Melastomataceae. Plastomes were assembled, annotated and compared to eight sequenced plastids in the Myrtales. Phylogenetic inference was performed using Maximum Likelihood on six different data sets, where putative biases were taken into account. Summary statistics were generated for all introns and intergenic spacers with suitable size for polymerase chain reaction (PCR) amplification and used to rank the markers by phylogenetic information.

**Results:**

The majority of the plastomes sampled are conserved in gene content and order, as well as in sequence length and GC content within plastid regions and sequence classes. Departures include the putative presence of *rps16* and *rpl2* pseudogenes in some plastomes. Phylogenetic analyses of the majority of the schemes analyzed resulted in the same topology with high values of bootstrap support. Although there is still uncertainty in some relationships, in the highest supported topologies only two nodes received bootstrap values lower than 95%.

**Discussion:**

Melastomataceae plastomes are no exception for the general patterns observed in the genomic structure of land plant chloroplasts, being highly conserved and structurally similar to most other Myrtales. Despite the fact that the full plastome phylogeny shares most of the clades with the previously widely used and reduced data set, some changes are still observed and bootstrap support is higher. The plastome data set presented here is a step towards phylogenomic analyses in the Melastomataceae and will be a useful resource for future studies.

## Introduction

The Melastomataceae Juss. has over 5,000 species distributed predominantly across the tropical regions. The observed levels of diversity, endemism or abundance of its members across different habitats make the family an important ecological group, as well as an excellent model for a variety evolutionary studies. The Melastomataceae belong in the Myrtales, where it is sister to the small CAP clade (Crypteroniaceae, Alzateaceae and Penaeaceae), which all together form a clade sister to Myrtaceae + Vochysiaceae (Berger et al., 2016). Plastid markers along with the nuclear ribosomal spacers (nrETS and nrITS) have been the major, and very often the exclusive, source of phylogenetic information in the family. Melastomataceae debut in molecular phylogenies was in a Myrtales-focused study, based on a partial amino acid sequence of the *rbcS* gene (Martin & Dowd, 1986). This study was followed by a more comprehensive nucleotide-based phylogeny, where the plastid *rbcL* gene was analyzed (Conti, Litt & Sytsma, 1996). The first Melastomataceae-wide phylogeny used a plastid data set including the *rbcL* and *ndhF* genes plus the *rpl16* intron (Clausing & Renner, 2001). This plastid data set is still the most employed source of information in studies focusing on generic relationships across the family (Fritsch et al., 2004; Renner, 2004; Amorim, Goldenberg & Michelangeli, 2009; Michelangeli et al., 2011; Goldenberg et al., 2012; Michelangeli, Ulloa & Sosa, 2014; Goldenberg et al., 2015; Zeng et al., 2016). Phylogenetic studies within lower lineages of Melastomataceae have predominantly used the plastid spacers *accD-psaI*, *atpF-atpH*, *psbK-psbI*, and *trnS-trnG*, along with the ribosomal spacers nrETS and nrITS (Bécquer-Granados et al., 2008; Reginato, Michelangeli & Goldenberg, 2010; Kriebel, Michelangeli & Kelly, 2015; Reginato & Michelangeli, 2016). Recently, the latter data set has also been used at deeper level studies (Michelangeli et al., 2013; Rocha et al., 2016).

Family-wide phylogenetic studies based on plastid markers have uncovered major relationships in the Melastomataceae, with several implications to the classification and evolutionary understanding in the family. Early studies have consolidated the sister relationship of Olisbeoideae and the remaining Melastomataceae, settling on the currently accepted family circumscription (Conti, Litt & Sytsma, 1996; Angiosperm Phylogeny Group, 1998; but see Clausing & Renner, 2001 for a different perspective). Latter studies focused in some tribal re-arrangements (Fritsch et al., 2004; Penneys et al., 2010; Michelangeli et al., 2011), generic placement (Amorim, Goldenberg & Michelangeli, 2009; Goldenberg et al., 2012; Michelangeli, Ulloa & Sosa, 2014; Goldenberg et al., 2015; Kriebel, 2016; Rocha et al., 2016; Zeng et al., 2016), phylogenetic evaluation of higher species-rich lineages (Michelangeli et al., 2004; Stone, 2006; Goldenberg et al., 2008; Martin et al., 2008; Michelangeli et al., 2008; Michelangeli et al., 2013), and lower taxon phylogenies (Bécquer-Granados et al., 2008; Reginato, Michelangeli & Goldenberg, 2010; Penneys, 2013; Kriebel, Michelangeli & Kelly, 2015; Gamba-Moreno & Almeda, 2014; Majure et al., 2015; Reginato & Michelangeli, 2016). Even in family-wide phylogenies, the level of variation across these few sampled plastid markers is unsatisfactory, as evidenced by low statistical support among many relationships in different published analyses. This issue becomes more prominent in phylogenetic analyses of lineages within Melastomataceae, where the plastid phylogeny is overall weakly supported, and concatenated results tend to be dominated by the more variable nuclear ribosomal data (Reginato, Michelangeli & Goldenberg, 2010; Reginato & Michelangeli, 2016).

Phylogenomic studies are sparse in the Myrtales and absent in the Melastomataceae. Currently, there are 54 full plastids of Myrtales on the NCBI database, covering three out of the nine families in the order (Lythraceae, Myrtaceae and Onagraceae). Full plastomes can potentially improve hypotheses of phylogenetic relationships within the family, as well as in the Myrtales, and provide basic information for other aspects of molecular biology (e.g., DNA barcoding, plastome evolution, development of molecular markers). Here, we present the first complete plastid genomes in the Melastomataceae, covering 16 species spread across the family. The objectives of this study are to describe the structure of the sampled plastomes; compare main features of the plastomes within the family and to other available Myrtales plastomes; and survey the plastomes for highly informative phylogenetic markers for future use.

## Material and Methods

### Taxon sampling, DNA extraction and sequencing

Genome skimming was performed for 16 species of Melastomataceae. Sampling was based on previous family wide phylogenetic studies (Michelangeli, Ulloa & Sosa, 2014; Goldenberg et al., 2015), where each sample belongs to a different major lineage of the family, either with a formal tribe status or not. Voucher information along with GenBank accession codes are presented in Table 1. Total genomic DNA was isolated from silica-dried tissue using the Qiagen DNAeasy plant mini-kit (Qiagen, Valencia, CA, USA) following the protocol suggested by Alexander et al. (2007) or used a modified CTAB extraction where the aqueous supernatant was silica-column purified (Neubig et al., 2014). Total DNA samples were quantified using a NanoDrop Spectrophotometer (Thermo Scientific, Waltham, MA, USA) or Qubit 2.0 (Invitrogen, Carlsbad, CA, USA). Total genomic libraries and barcoding was performed at Cold Spring Harbor Laboratories or at Rapid Genomics (Gainesville, FL, USA) for sequencing on an Illumina HiSeq2000 platform (Illumina, Inc., San Diego, CA, USA).

**Table 1:** Voucher information and GenBank accessions of the chloroplast sequenced in the Melastomataceae.

Species	Tribe/“clade”	GenBank	Voucher	Herbarium
*Allomaieta villosa* (Gleason) Lozano	Cyphostyleae	KX826819	David, H. 2188	HUA, NY
*Bertolonia acuminata* Gardner	Bertolonieae	KX826820	Goldenberg, R. 810	NY, UPCB
*Blakea schlimii* (Naudin) Triana	Blakeeae	KX826821	Michelangeli, F.A. 1227	NY
*Eriocnema fulva* Naudin	“Eriocnema”	KX826822	Almeda, F. 8416	CAS
*Graffenrieda moritziana* Triana	Merianieae	KX826823	Michelangeli, F.A. 832	NY
*Henriettea barkeri* (Urb. & Ekman) Alain	Henrietteeae	KX826824	Ionta, G. 2029	FLAS
*Merianthera pulchra* Kuhlm.	“Cambessedesia”	KX826825	Goldenberg, R. 1153	NY, UPCB
*Miconia dodecandra* Cogn.	Miconieae	KX826826	Michelangeli, F.A. 758	NY
*Nepsera aquatica* (Aubl.) Naudin	“Marcetia”	KX826827	Michelangeli, F.A. 1998	NY
*Opisthocentra clidemioides* Hook. f.	Unplaced	KX826828	Caddah, M.K. 578	NY, UPCB
*Pterogastra divaricata* (Bonpl.) Naudin	Melastomeae	KX826829	Michelangeli, F.A. 540	NY
*Rhexia virginica* L.	Rhexieae	KX826830	Michelangeli, F.A. 1448	NY
*Rhynchanthera bracteata* Triana	Microlicieae	KX826831	Zenteno, F. 8801	NY
*Salpinga maranoniensis* Wurdack	Merianieae	KX826832	Clark, J.L. 13577	UNA
*Tibouchina longifolia* (Vahl) Baill.	Melastomeae	KX826833	Majure, L. 4277	FLAS
*Triolena amazonica* (Pilg.) Wurdack	“Triolena”	KX826834	Michelangeli, F.A. 1366	NY

**Note:**

Informal clades are quoted.

### Plastid genome assembly and annotation

Total reads number yielded was on average ca. 11.5 Gb per sample (s.d. = 6 Gb). Paired reads were imported into Geneious 7.1 (Biomatters Ltd., Auckland, New Zealand), trimmed by quality (at 0.05 probability) and de novo assembled (Geneious Assembler, “low sensitivity” option, default settings). Filtered assembled contigs (length > 1 kb) were blasted against the *Eucalyptus polybractea* plastome (NC022393). The identified plastid contigs were then reference assembled against the *E. polybractea* plastome in order to generate a single contig to construct the circular maps. Eventual short gaps were filled by iteratively mapping the total paired reads against the contig ends. Plastid annotation was performed in Geneious 7.1 with *Arabidopsis thaliana* (NC000932) and *Eucalyptus polybractea* (NC022393) as references. Graphical representations of the plastid circular and linear maps were generated with OGDRAW (Lohse et al., 2013) and the R package genoPlotR (R Development Core Team, 2016; Guy, Kultima & Andersson, 2010).

Plastome structure, gene content, and general characteristics of the plastid genome were compared among the 16 Melastomataceae plastomes and to eight published plastomes of Myrtales, covering all families in this order available on the NCBI website. The Myrtales plastomes included one species in the Lythraceae (*Lagerstroemia fauriei*–NC029808), one Onagraceae (*Oenothera grandiflora*–NC029211) and six Myrtaceae (*Allosyncarpia ternata*–NC022413; *Angophora costata*–NC022412; *Corymbia gummifera*–NC022407; *Eucalyptus polybractea*–NC022393; *Eugenia uniflora*–NC027744; and *Stockwellia quadrifida*–NC022414).

### Phylogenetic analyses

Three major data sets were generated for phylogenetic inference. The first included the non-coding regions (ncs data set), the second included 78 protein-coding genes (cds data set), and the third consisted of fully assembled plastomes (full data set). In all data sets one of the IR sequences was removed to reduce overrepresentation of duplicated sequences. Full plastids were aligned with MAFFT v. 7 using the FFT-NS-i × 1,000 strategy (Katoh & Standley, 2013). Coding sequences were extracted from the full alignment, resulting in the cds and ncs data sets. Each gene in the cds data set was re-aligned using its translation under the same strategy of the full data set and then concatenated. Given that phylogenetic inference might be biased by poorly aligned regions with ambiguous homology, heterogeneous rates of substitution in the different codon positions, synonymous substitutions in Arginine, Leucine and Serine codons, among others (Misof & Misof, 2009; Cox et al., 2014), we further divided the three major data sets into six different schemes where we attempted to circumvent those issues. Poorly aligned regions of the ncs data set were removed using aliscore.pl with the −N and −r options (Misof & Misof, 2009), and in the cds data set; all codons coding for Arginine, Leucine and Serine were ambiguated. Thus, the final six schemes included: 1. all ncs data set (ncs); 2. ncs data set without poorly aligned sites (ncs filtered); 3. all cds data set (cds); 4. cds with A, L and S codons ambiguated (cds ambiguated); 5. translated cds (protein); 6. ncs filtered plus all cds non-ambiguated (full). Additionally, in order to carry out a more objective comparison with previous phylogenetic hypotheses, we also analyzed a reduced data set that included only the three more commonly used markers for family wide phylogenies in the Melastomataceae (*ndhF* and *rbcL* genes along with the *rpl16* intron, concatenated).

Phylogenetic inference for all schemes was performed using Maximum Likelihood implemented in RAxML 8.2.4 (Stamatakis, 2014). The GTR+G model was employed for all nucleotide data and the PROT+G model for the protein sequences. Support was estimated through 1,000 bootstrap replicates. Protein-coding sequences were partitioned by codon position in all schemes, while no partitioning was employed for the non-coding regions.

### Phylogenetic informative regions

In order to identify and rank highly phylogenetically informative regions in the Melastomataceae plastomes, all introns (19) and variable intergenic spacers with suitable size for PCR amplification (22) were selected and compared. Each individual marker was aligned with MAFFT (FFT-NS-i × 1,000 strategy), and its Maximum likelihood tree inferred with RAxML (not partitioned, GTR+G model, 100 bootstrap replicates). For each marker, we report the number of variable sites, number of parsimony informative sites, mean sequence distance (under K80 model), alignment length, mean sequence length, mean bootstrap support and distance to the full scheme plastid tree (RF distance; Robinson & Foulds, 1981). The metrics were retrieved using functions of the R packages ape and phangorn (Paradis, Claude & Strimmer, 2004; Schliep, 2011). Markers were ranked by phylogenetic information using a weighted mean of relative values of the following metrics: number of variable sites (weight = 1), mean bootstrap (weight = 2) and distance to the full plastid tree (weight = 3). For the top 10 markers identified in the previous step, we designed primer pairs for PCR amplification. Primers flanking the target regions were designed with Primer3, using the default settings (Rozen & Skaletsky, 2000). All metrics reported, as well primer design, considered only the ingroup (the 16 Melastomataceae plastids).

## Results

### Plastome structure

**Figure 1:**
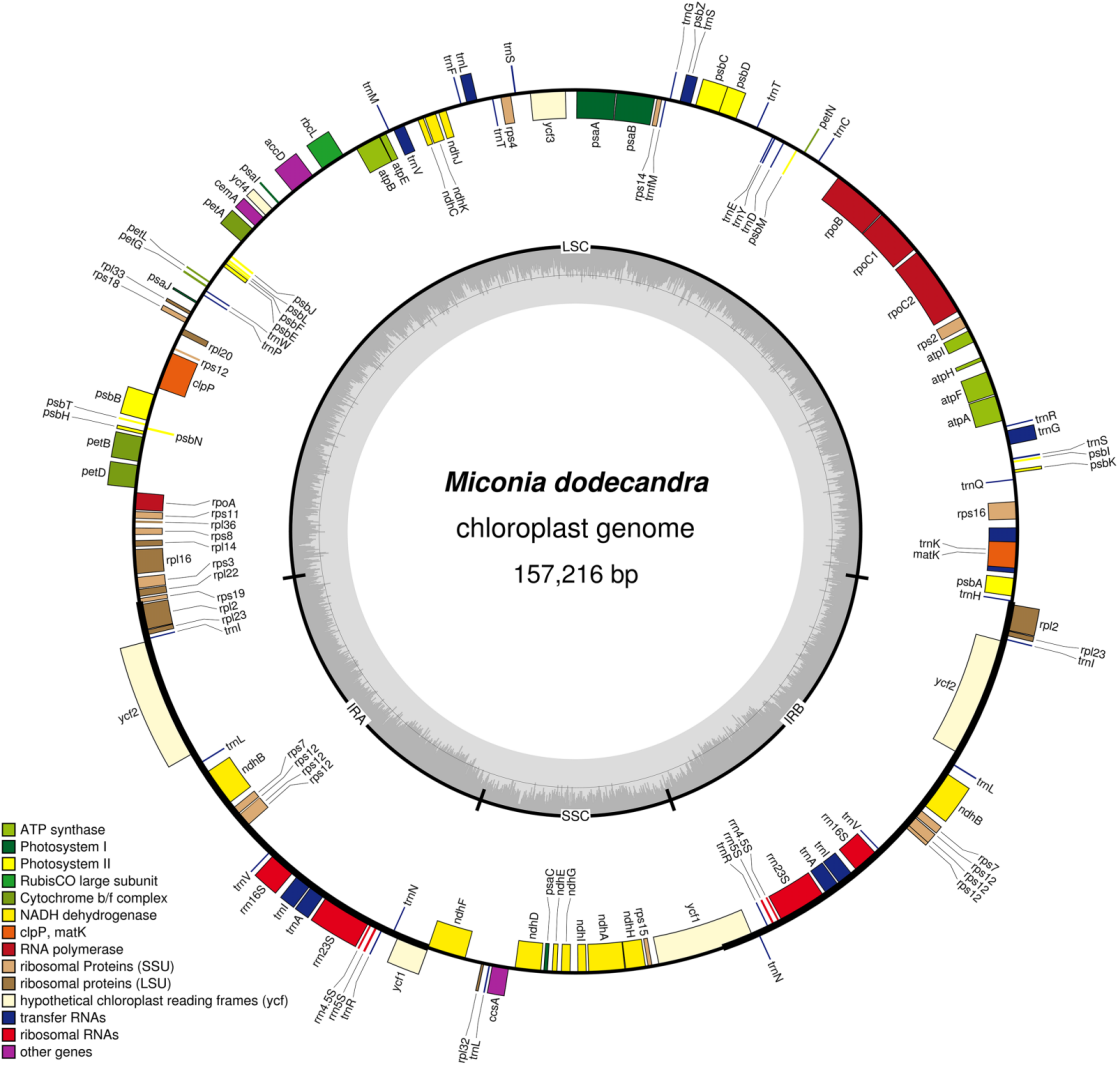
Map of the *Miconia dodecandra* plastid genome. Genes shown outside the outer circle are transcribed counterclockwise and genes inside the outer circle are transcribed clockwise. Genes in different functional groups are color coded following the legend. The shaded area inside the inner circle indicates the GC content, with dark shading indicating GC percent.

**Figure 2:**
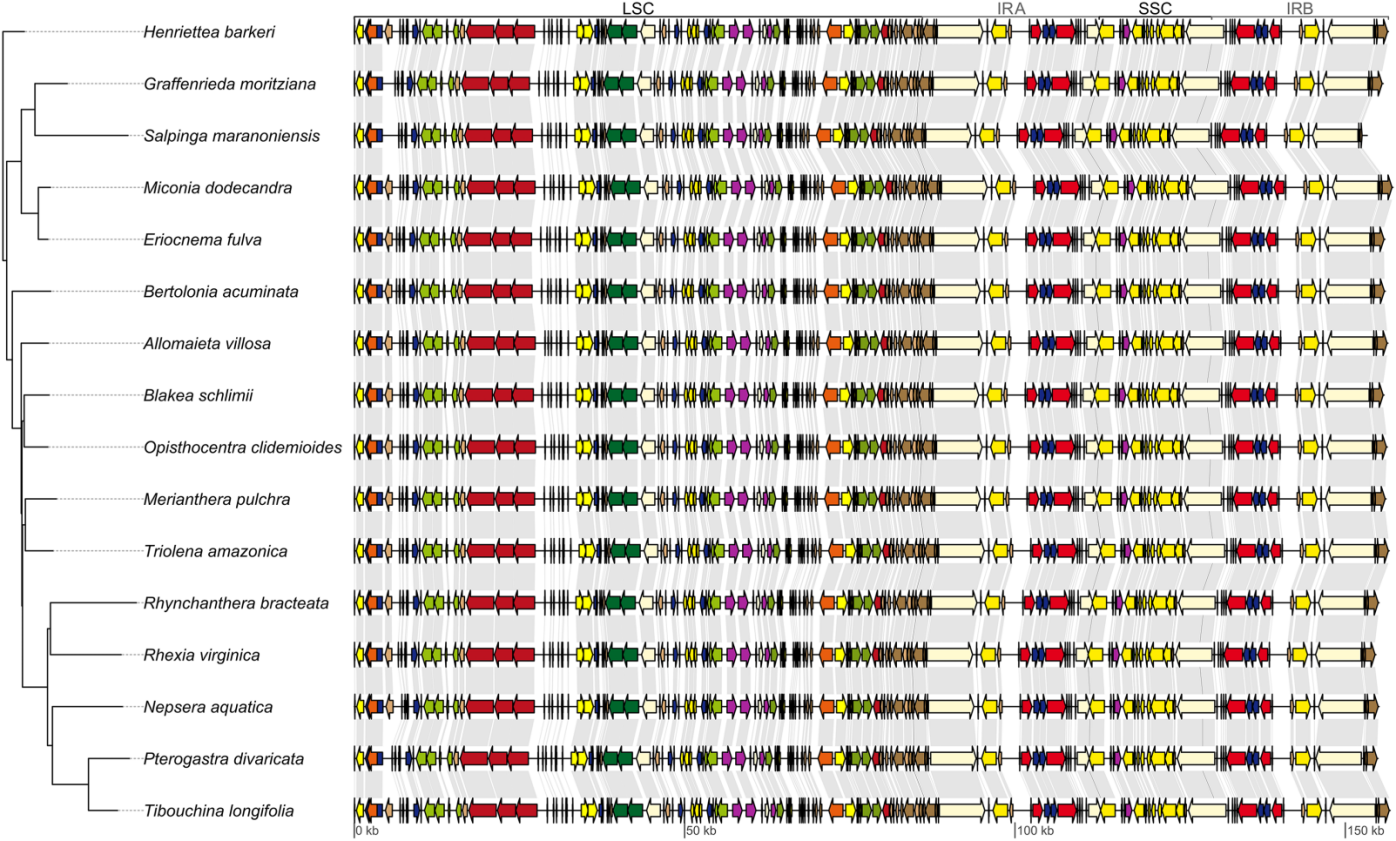
Maximum likelihood tree recovered with the full data set (left). On the right, linear plastid maps of the 16 Melastomataceae species. All genes are depicted as arrows (indicating transcription direction) and color coded following the legend of Fig. 1. Gray lines link the same genes on contiguous maps. LSC, long single copy region; SSC, small single copy region; IRA, inverted repeat A; IRB, inverted repeat B.

All plastomes have a quadripartite organization, with one large single copy region (LSC), one small single copy (SSC) and two inverted repeats (IRs). A circular map of the *Miconia dodecandra* plastome is presented in Fig. 1 and linear maps of all Melastomataceae plastomes in Fig. 2. Sequence depth ranged from 42 to 705 (mean = 289) and plastome length from 153,311 to 157,216 bp (mean = 155,806 pb). Sequence length and GC content of the different regions across the Melastomataceae plastomes are presented in Table 2. Overall, GC content is similar across species within the same plastid region, while the LSC regions has the greatest standard deviation in sequence length (s.d. = 616 bp), followed by IR (s.d. = 250 bp) and the SSC (s.d. = 126 bp).

**Table 2:** Comparison of plastid genome size and GC content across different regions in the 16 Melastomataceae species.

Species	Coverage (mean)	LSC		SSC		IR		Full	
		bp	GC	bp	GC	bp	GC	bp	GC
*Allomaieta villosa*	278	85,915	0.347	16,975	0.306	26,781	0.425	156,452	0.369
*Bertolonia acuminata*	189	85,571	0.347	17,008	0.308	26,733	0.425	156,045	0.370
*Blakea schlimii*	170	85,370	0.349	16,998	0.308	26,747	0.425	155,862	0.370
*Eriocnema fulva*	42	85,431	0.348	16,953	0.308	26,805	0.425	155,994	0.370
*Graffenrieda moritziana*	683	85,341	0.347	16,924	0.309	26,734	0.425	155,733	0.370
*Henriettea barkeri*	130	85,991	0.347	17,036	0.306	26,750	0.425	156,527	0.369
*Merianthera pulchra*	56	85,621	0.348	17,001	0.307	26,773	0.424	156,168	0.370
*Miconia dodecandra*	318	86,609	0.348	16,999	0.310	26,804	0.425	157,216	0.370
*Nepsera aquatica*	705	84,644	0.348	17,066	0.310	26,700	0.426	155,110	0.371
*Opisthocentra clidemioides*	100	85,866	0.348	16,942	0.309	26,772	0.425	156,352	0.370
*Pterogastra divaricata*	184	84,718	0.351	17,156	0.312	26,537	0.425	154,948	0.372
*Rhexia virginica*	683	84,459	0.351	16,924	0.311	26,626	0.425	154,635	0.372
*Rhynchanthera bracteata*	304	85,093	0.347	16,729	0.307	26,643	0.426	155,108	0.370
*Salpinga maranoniensis*	537	85,128	0.353	16,653	0.317	25,765	0.428	153,311	0.374
*Tibouchina longifolia*	195	86,297	0.349	17,124	0.311	26,684	0.425	156,789	0.371
*Triolena amazonica*	48	86,200	0.347	16,970	0.307	26,741	0.425	156,652	0.369

**Notes:**

Length, length in bp; GC, GC content %.

LSC, long single copy region; SSC, small single copy region; IR, inverted repeat; Full, full plastome.

Most plastomes have 84 protein-coding genes (CDS), 37 transfer RNA (tRNA) and eight ribosomal (rRNA), totaling 129 genes (including duplicates and *ycf1*, *ycf2*, *ycf3* and *ycf4*). Among the duplicated genes in the IR, there are six CDS, seven tRNA, and four rRNA. As for the plastid regions, GC content is similar across different species within the same sequence class (CDS, tRNA, rRNA, intron and intergenic spacers), whereas the greatest variation in sequence length is observed across intergenic spacers (s.d. = 617 bp). A comparative summary of length and GC content in the different sequence classes across the Melastomataceae plastomes is given in Table 3. In the majority of the species sampled, gene content and order is similar to other Myrtales plastids, such as *Lagerstroemia fauriei* (NC029808) and *Eucalyptus polybractea* (NC022393). The exceptions are *rps16* and *rpl2*, which are putative pseudogenes in some plastids. The former seems to have been pseudogenized in *Graffenrieda moritziana* and *Pterogastra divaricata* (where the first exon is absent) and in *Salpinga margaritacea* (with several insertions changing the reading frame in the second exon); the second copy of *rpl2* gene (in the IRB) is likely a pseudogene in *Salpinga margaritacea* due to a shift in the IRB-LSC boundary in that plastid, which resulted in the loss of the second exon. Additionally, some variation is observed in all region boundaries across the Melastomataceae plastomes. The LSC-IRA boundary is located in the *rps19* gene in most species, except in *S. margaritacea* where it is located in the intron of the *rpl2* gene; the IRA-SSC boundary is located in the overlapping ψ*ycf1* and *ndhF*; the SSC-IRB in the *ycf1*; and the IRB-LSC in the *rpl2*-*trnH* spacer or in the *trnH* gene. Introns are found in 17 genes in all Melastomataceae plastomes, including six tRNA genes and 11 CDS, from which three have two introns (*clpP*, *rps12* and *ycf3*). A comparison of the number of genes, regions and plastome length of one Melastomataceae (*M. dodecandra*) and eight Myrtales plastids is presented in Table 4. The sequence length of the full plastome and its regions in the Melastomataceae sampled here are in the range observed for other Myrtales.

**Table 3:** Comparison of length and GC content across different sequence classes in the plastome of the 16 Melastomataceae species.

Species	Protein-coding		tRNA		rRNA		Intron		Intergenic	
	bp	GC	bp	GC	bp	GC	bp	GC	bp	GC
*Allomaieta villosa*	80,826	0.374	3,348	0.497	9,050	0.425	20,553	0.347	42,675	0.316
*Bertolonia acuminata*	80,670	0.375	3,356	0.497	9,050	0.425	20,437	0.347	42,532	0.316
*Blakea schlimii*	80,742	0.375	3,348	0.498	9,050	0.425	20,541	0.347	42,181	0.319
*Eriocnema fulva*	80,628	0.375	3,354	0.497	9,050	0.425	20,540	0.347	42,422	0.318
*Graffenrieda moritziana*	80,286	0.375	3,349	0.497	9,050	0.425	19,691	0.347	43,357	0.317
*Henriettea barkeri*	80,781	0.374	3,363	0.495	9,050	0.425	20,571	0.347	42,762	0.315
*Merianthera pulchra*	80,751	0.375	3,364	0.498	9,050	0.425	20,478	0.347	42,525	0.318
*Miconia dodecandra*	80,586	0.376	3,354	0.498	9,050	0.425	20,548	0.347	43,678	0.317
*Nepsera aquatica*	80,646	0.375	3,370	0.496	9,050	0.425	20,619	0.347	41,425	0.318
*Opisthocentra clidemioides*	80,643	0.376	3,360	0.496	9,050	0.425	20,641	0.347	42,658	0.317
*Pterogastra divaricata*	80,427	0.377	3,339	0.498	9,050	0.425	19,911	0.347	42,221	0.318
*Rhexia virginica*	80,466	0.377	3,353	0.496	9,050	0.425	20,260	0.347	41,506	0.319
*Rhynchanthera bracteata*	80,415	0.375	3,241	0.502	9,048	0.425	20,538	0.347	41,866	0.317
*Salpinga maranoniensis*	79,326	0.376	3,349	0.500	9,050	0.425	18,991	0.347	42,595	0.326
*Tibouchina longifolia*	80,682	0.377	3,348	0.497	9,050	0.425	20,666	0.347	43,043	0.317
*Triolena amazonica*	80,619	0.375	3,337	0.496	9,050	0.425	20,476	0.347	43,170	0.316

**Note:**

Length, length in bp; GC, GC content %.

**Table 4:** Comparison of plastid genome size of one Melastomataceae species (*Miconia dodecandra*) with eight other Myrtales.

Family	Species	Coding	tRNA	rRNA	LSC	SSC	IR	Full
Melastomataceae	*Miconia dodecandra*	84	37	8	86,609	16,999	26,804	157,216
Myrtaceae	*Allosyncarpia ternata*	84	37	8	88,218	18,571	26,402	159,563
Myrtaceae	*Angophora costata*	84	37	8	88,769	18,773	26,392	160,326
Myrtaceae	*Corymbia gummifera*	84	37	8	88,310	17,197	27,603	160,713
Myrtaceae	*Eucalyptus polybractea*	84	37	8	88,944	18,530	26,397	160,268
Myrtaceae	*Eugenia uniflora*	84	37	8	87,459	18,318	26,334	158,445
Lythraceae	*Lagerstroemia fauriei*	84	37	8	83,923	16,933	25,792	152,440
Onagraceae	*Oenothera grandiflora*	84	38	8	89,862	19,035	28,824	166,545
Myrtaceae	*Stockwellia quadrifida*	84	37	8	88,247	18,544	26,385	159,561

**Notes:**

Protein-coding, tRNA and rRNA (number of genes); LSC, long single copy region, length in bp; SSC, small single copy region, length in bp; IR, inverted repeat, length in bp and Full (length in bp).

### Phylogenetic analyses

**Figure 3:**
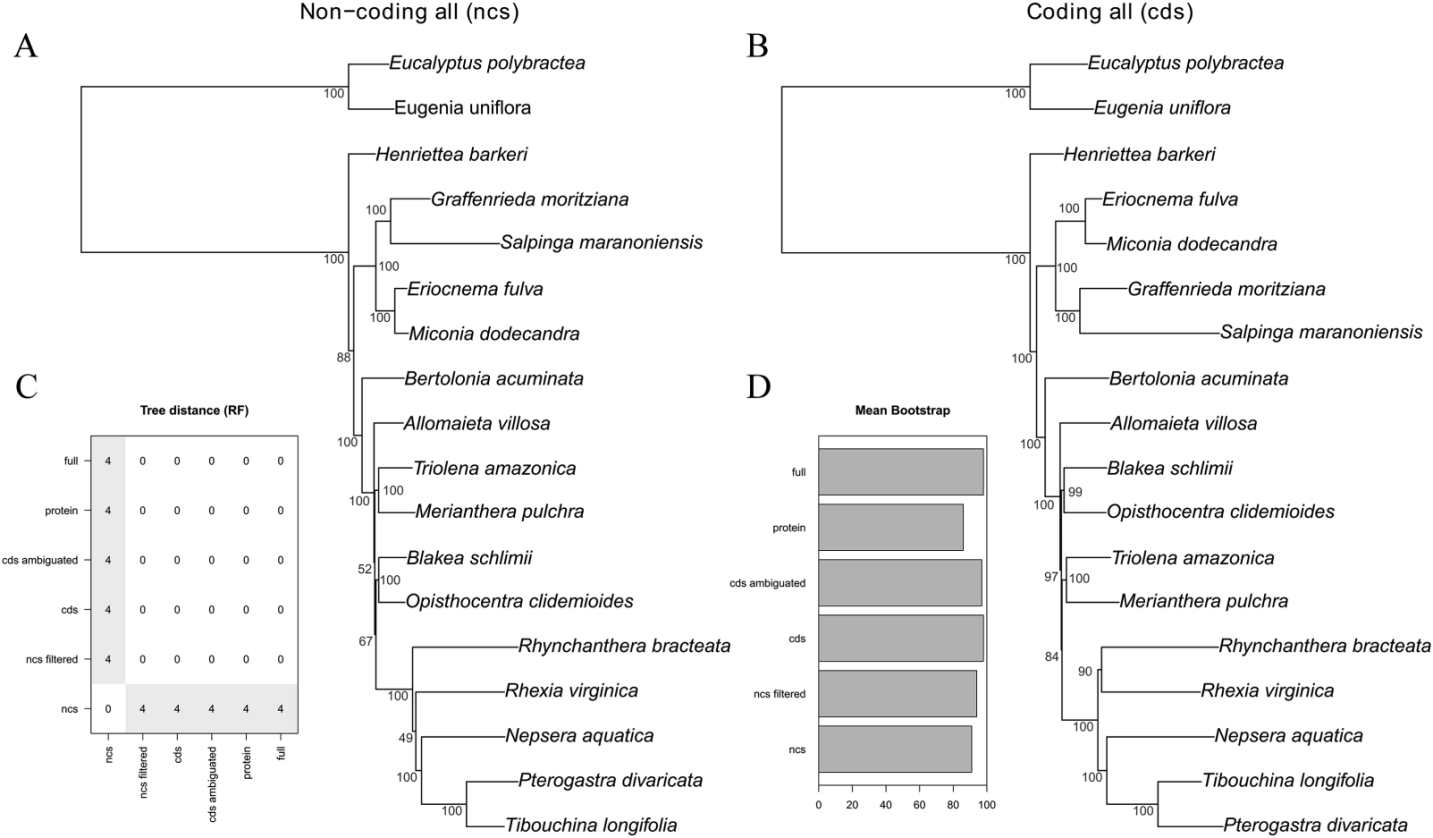
Maximum likelihood trees of the all non-coding–ncs (A) and all coding genes–cds (B) data sets. Bootstrap support is given adjacent to the nodes. (C) Tree distance (RF) pairwise matrix between all six schemes analyzed. (D) Mean bootstrap support of all six schemes analyzed.

The majority of the six analytical schemes recovered the same topology (Figs. 2 and 3B). The only exception was the “all non-coding” scheme (i.e., the full non-coding regions without filtering of dubiously aligned base pairs), where *Blakea* + *Opistocentra*, *Triolena* + *Merianthera* and *Rhynchanthera* assume a different position (Fig. 3A). Pairwise tree distances among all schemes are depicted in Fig. 3C, and all Maximum Likelihood trees with bootstrap support values are given in the Fig. S1. Bootstrap support is highest in the “full” and “cds” schemes and lower in the “protein” and “all non-coding” schemes (Fig. 3D). In the highest supported topologies, there are only two nodes with bootstrap values lower than 95, and those involve the relationship disagreements between the two alternate topologies (Figs. 3A and 3B). While filtering the non-coding poorly aligned sites improved bootstrap support and also changed the topology (“ncs” vs. “ncs filtered,” Fig. 3), ambiguating common amino acids in the coding sequences did not have any apparent effect in the topology or support values (“cds” vs. “cds ambiguated;” Fig. 3D).

The commonly used plastid data set in previous family-wide studies (*rbcL*, *ndhF* and *rpl16* intron) also resulted in a different topology from the “full” scheme, although with most clades in common (Fig. S2). Disagreements involved the position of *Allomaieta*, *Trioleta* + *Merianthera*, *Blakea* + *Opisthocentra*, and *Rhynchanthera*; these disagreements manifest in nodes of low bootstrap support where, in the reduced data set, they range from 24 to 100 (mean = 73).

### Phylogenetically informative regions

Summary statistics for all intron and intergenic spacers with suitable size for PCR amplification are presented in Table S1. A list of the top 10 markers ranked by phylogenetic information, taking into account topological distance to the tree based on the “full” scheme (Fig. 2), mean bootstrap support and number of variable sites is given in Table 5, and the full list is available in Table S1. All single marker phylogenies presented some disagreement to the tree based on the “full” scheme (RF tree distance ranging from 4 to 22). Bootstrap support ranged from 26 to 82 (mean = 63) and number of variable sites from 12 to 507 (mean = 224). Primer pair sequences for PCR amplification are provided for the top 5 markers in Table 6.

**Table 5:** Summary statistics for the top 10 introns and intergenic spacers with suitable size for PCR amplification. Markers are ranked by phylogenetic information based on a weighed mean of relative values of number of variable sites (weight = 1), mean bootstrap (weight = 2) and distance to the full plastid tree (weight = 3).

Marker	Bases	Aligned (bp)	Variable sites	PIS	DNA distance (mean)	Tree distance	Bootstrap (mean)
1. *trnS-trnG* spacer	780 [628, 884]	1,125	438 (38.9%)	128 (11.4%)	0.104	4	82
2. *ndhF-rpl32* spacer	898 [849, 965]	1,266	507 (40%)	171 (13.5%)	0.114	6	71
3. *trnG* intron	762 [743, 790]	846	236 (27.9%)	76 (9%)	0.059	4	75
4. *ndhC-trnV* spacer	734 [504, 821]	991	330 (33.3%)	98 (9.9%)	0.081	4	63
5. *ndhA* intron	1,016 [939, 1,045]	1,127	250 (22.2%)	74 (6.6%)	0.046	4	64
6. *trnG-atpA* spacer	641 [550, 750]	895	353 (39.4%)	136 (15.2%)	0.114	6	65
7. *atpH-atpI* spacer	898 [638, 980]	1,178	323 (27.4%)	92 (7.8%)	0.062	8	76
8. *psbE-petL* spacer	1,058 [570, 1,165]	1,396	381 (27.3%)	132 (9.5%)	0.068	8	70
9. *petA-psbJ* spacer	736 [420, 944]	1,062	285 (26.8%)	90 (8.5%)	0.076	8	76
10. *trnE-trnT* spacer	842 [478, 1,029]	1,345	406 (30.2%)	121 (9%)	0.089	8	63

**Note:**

PIS, parsimony informative sites; Tree distance, RF distance.

**Table 6:** Primer pair sequences for the identified top five highly informative markers across the 16 plastomes of Melastomataceae.

Marker	Primer forward (5′–3′)	Primer reverse (5′–3′)	T_a_ (°C)
1. *trnS-trnG* spacer	CACTCAGCCATCTCTCCCAA	ACCCGCTACAATGCCATTATTG	55
2. *ndhF-rpl32* spacer	AGGAAAGGACCACATACGTCG	TCCTTGCTCATTGATTTTGATCCA	55
3. *trnG* intron	GGTCCCTCGGATTTGCTTCA	GAACCCGCATCGTTAGCTTG	55
4. *ndhC-trnV* spacer	AGATGAACTCCTAGGGAATGTGA	CCGAGAAGGTCTACGGTTCG	55
5. *ndhA* intron	CGCTAGTCCAGAACCGTACA	ACCCCATGATTGGTTGATTAGTGA	55

## Discussion

Plastid genomes of higher plants are of relatively small size, ranging from 115 to 165 kb in most groups, with an average of 90 CDS across most land plants (Ravi et al., 2008; Wicke et al., 2011). In general, the quadripartite organization, gene content and order are conserved, and GC content is usually stable within plastid regions and sequence classes (Ravi et al., 2008; Wicke et al., 2011). Melastomataceae plastomes are no exception for these patterns, being highly conserved and structurally similar to most other Myrtales, as well as to an ordinary angiosperm plastome. Melastomataceae plastomes’ mean length (156 kb) is closer to the upper bound observed across most plants (165 kb), while the number of genes and GC content are around the average (90 genes, GC = 37%; Ravi et al., 2008). High conservation in genomic structure of plastomes among the Myrtales has been previously suggested (Gu et al., 2016) and is extended here to include Melastomataceae. The greatest variation in sequence length among different region classes in Melastomataceae are observed in the intergenic spacers, which is also another general pattern in plastomes (Ravi et al., 2008; Gu et al., 2016). Additionally, the boundaries of the IRs vary, as observed in some Myrtales and other groups (Bayly et al., 2013).

Conservation in gene order, content and virtual lack of recombination make the plastome a useful tool for plant phylogenetic studies (Ravi et al., 2008). An updated comprehensive phylogenetic hypothesis for the entire Melastomataceae is overdue, and full plastid sequences would contribute greatly to such an endeavor. Additionally, as sampling increases in the Myrtales, full plastids also might help to narrow down phylogenetic uncertainty in the Myrtales (e.g., Combretaceae position, Berger et al., 2016). Despite the fact that the full plastome phylogeny recovered here shares most of the clades with the widely used “*rbcL* + *ndhF* + *rpl16*” tree, some changes are still observed and bootstrap support is higher. A more conclusive account on the extent of such changes will require more taxa to be sampled.

Here, we provide a list of potentially highly informative plastid markers for Melastomataceae. We acknowledge that the information descriptors employed are very sensitive to the taxa under analysis. Nonetheless, this ranked list can be used as guidance for sampling design of future studies, whereas the new family specific primers will increase the plastid options for Sanger sequencing-based phylogenies. There has been some debate as to whether the availability of full plastome sequencing (and other NGS tools) would render Sanger sequencing obsolete (Hert, Fredlake & Barron, 2008). Here we show that a full plastome phylogeny is an improvement on single or few plastid loci phylogenies, especially on the level of statistical support. However, considering scalability, computational complexity and budget limitations, a comprehensive NGS-based phylogeny for the mega-diverse Melastomataceae might not be achieved in the short term. Nonetheless, an expanded full plastome data set along with the more abundant Sanger-based sequences available, could be coupled in future studies. A hybrid NGS and Sanger sequencing approach has been employed for other groups (Xi et al., 2012; Leaché et al., 2014; Gardner et al., 2016), and could help clarifying the backbone of a comprehensive Melastomataceae phylogeny. Recalcitrant phylogenetic backbones are a widespread and challenging phenomenon in angiosperms (Xi et al., 2012; Straub et al., 2014), and their resolution is critical to increase the confidence of ancestral state reconstructions, historical biogeographical scenarios and other evolutionary hypotheses. Although full plastomes, or an expanded sample of plastid markers, may help to improve the confidence of phylogenetic relationships within the Melastomataceae, we also recognize the need of parallel sampling of additional independent genealogies (i.e., nuclear and mitochondrial genomes) for further refinement in the Melastomataceae tree.

## Supplemental Information

10.7717/peerj.2715/supp-1 Supplemental Information 1 Figure S1. Maximum likelihood trees of all six analyzed schemes in this study. Bootstrap support is given adjacent to the nodes.

10.7717/peerj.2715/supp-2 Supplemental Information 2 Figure S2. Comparison of the Maximum Likelihood tree of the full data set (ncs filtered + cds; on the left) with a reduced data set of commonly used markers for family wide phylogenies in the Melastomataceae (*ndhF*, *rbcL* and *rpl16* intron; on the right). Bootstrap support is given adjacent to the nodes.

10.7717/peerj.2715/supp-3 Supplemental Information 3 Table S1. Summary statistics for all introns and intergenic spacers with suitable size for PCR amplification. Markers are ranked by phylogenetic information based on a weighed mean of relative values of number of variable sites (weight = 1), mean bootstrap (weight = 2) and distance to the full plastid tree (weight = 3). PIS = parsimony informative sites; Tree distance = RF distance; NA = not applicable.

10.7717/peerj.2715/supp-4 Supplemental Information 4 Annotated sequences of the 16 full plastomes of Melastomataceae.

## Additional Information and Declarations

### DNA Deposition

The following information was supplied regarding the deposition of DNA sequences: GenBank: KX826819; KX826820; KX826821; KX826822; KX826823; KX826824; KX826825; KX826826; KX826827; KX826828; KX826829; KX826830; KX826831; KX826832; KX826833; KX826834

### Data Deposition

The following information was supplied regarding data availability: The raw data has been supplied as Supplemental Dataset Files.
