# Supplementary figures and images for: The first complete plastid genomes of Melastomataceae are highly structurally conserved
Source: PeerJ. 2016 Nov 29;4:e2715. doi: 10.7717/peerj.2715 (PMC5131623; doi:10.7717/peerj.2715)

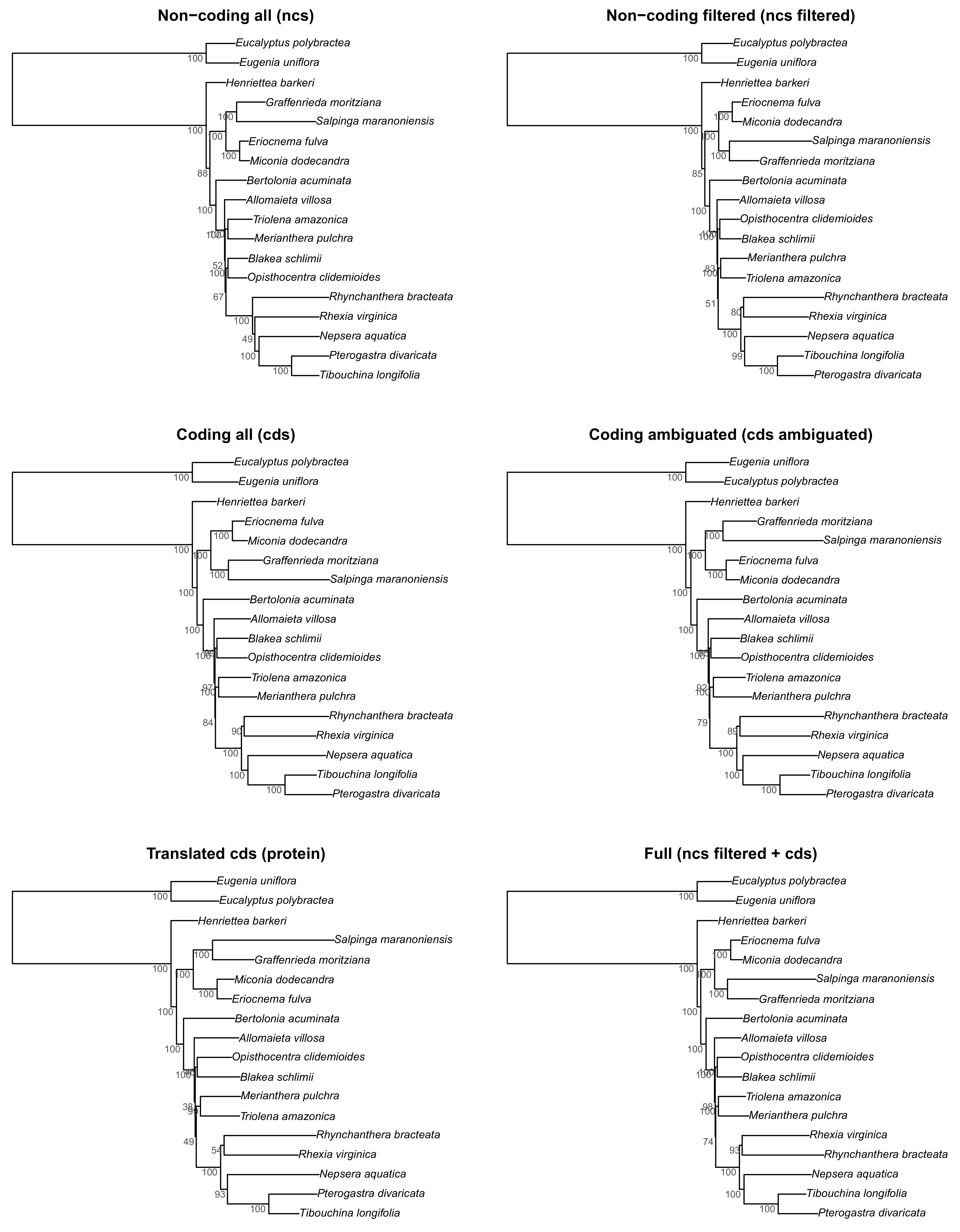

Supplement: Supplemental Information 1 — Maximum likelihood trees of all six analyzed schemes in this study. Bootstrap support is given adjacent to the nodes. [file peerj-04-2715-s001.png]

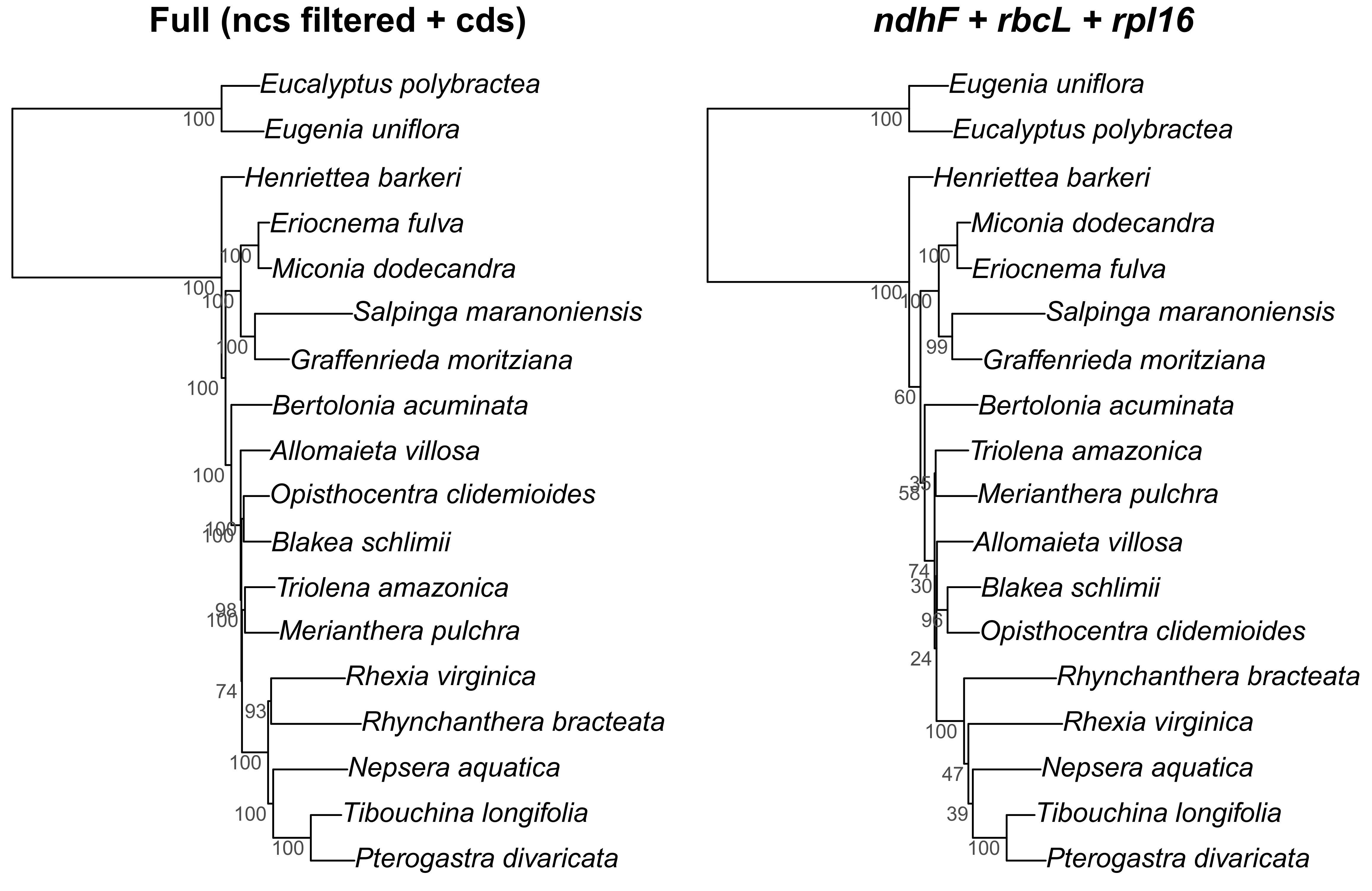

Supplement: Supplemental Information 2 — Comparison of the Maximum Likelihood tree of the full data set (ncs filtered + cds; on the left) with a reduced data set of commonly used markers for family wide phylogenies in the Melastomataceae (ndhF, rbcL and rpl16 intron; on the right). Bootstrap support is given adjacent to the nodes. [file peerj-04-2715-s002.png]
