# Supplementary material for: The first complete plastid genomes of Melastomataceae are highly structurally conserved
Source: PeerJ. 2016 Nov 29;4:e2715. doi: 10.7717/peerj.2715 (PMC5131623; doi:10.7717/peerj.2715)
Supplement: Supplemental Information 3 — Summary statistics for all introns and intergenic spacers with suitable size for PCR amplification. Markers are ranked by phylogenetic information based on a weighed mean of relative values of number of variable sites (weight = 1), mean bootstrap (weight = 2) and distance to the full plastid tree (weight = 3). PIS = parsimony informative sites; Tree distance = RF distance; NA = not applicable. [file peerj-04-2715-s003.docx]

| **Marker** | **Bases** | **Aligned Bases** | **Variable sites** | **PIS** | **DNA distance (mean)** | **Tree distance** | **Bootstrap (mean)** | **Taxa** |
| --- | --- | --- | --- | --- | --- | --- | --- | --- |
| 1. *trnS-trnG* spacer | 780 [628,884] | 1125 | 438 (38.9 %) | 128 (11.4 %) | 0.104 | 4 | 82 | 16 |
| 2. *ndhF-rpl32* spacer | 898 [849,965] | 1266 | 507 (40 %) | 171 (13.5 %) | 0.114 | 6 | 71 | 16 |
| 3. *trnG* intron | 762 [743,790] | 846 | 236 (27.9 %) | 76 (9 %) | 0.059 | 4 | 75 | 16 |
| 4. *ndhC-trnV* spacer | 734 [504,821] | 991 | 330 (33.3 %) | 98 (9.9 %) | 0.081 | 4 | 63 | 16 |
| 5. *ndhA* intron | 1016 [939,1045] | 1127 | 250 (22.2 %) | 74 (6.6 %) | 0.046 | 4 | 64 | 16 |
| 6. *trnG-atpA* spacer | 641 [550,750] | 895 | 353 (39.4 %) | 136 (15.2 %) | 0.114 | 6 | 65 | 16 |
| 7. *atpH-atpI* spacer | 898 [638,980] | 1178 | 323 (27.4 %) | 92 (7.8 %) | 0.062 | 8 | 76 | 16 |
| 8. *psbE-petL* spacer | 1058 [570,1165] | 1396 | 381 (27.3 %) | 132 (9.5 %) | 0.068 | 8 | 70 | 16 |
| 9. *petA-psbJ* spacer | 736 [420,944] | 1062 | 285 (26.8 %) | 90 (8.5 %) | 0.076 | 8 | 76 | 16 |
| 10. *trnE-trnT* spacer | 842 [478,1029] | 1345 | 406 (30.2 %) | 121 (9 %) | 0.089 | 8 | 63 | 16 |
| 11. *tRNA-trnL* spacer | 818 [600,872] | 1017 | 306 (30.1 %) | 109 (10.7 %) | 0.075 | 8 | 71 | 16 |
| 12. *trnD-trnE* spacer | 615 [587,686] | 779 | 246 (31.6 %) | 99 (12.7 %) | 0.076 | 8 | 69 | 16 |
| 13. *trnF-ndhJ* spacer | 855 [637,904] | 1035 | 329 (31.8 %) | 158 (15.3 %) | 0.101 | 10 | 71 | 16 |
| 14. *petD* intron | 809 [787,833] | 884 | 191 (21.6 %) | 65 (7.4 %) | 0.047 | 8 | 68 | 16 |
| 15. *clpP* intron 1 | 922 [727,964] | 1130 | 332 (29.4 %) | 100 (8.8 %) | 0.063 | 12 | 76 | 16 |
| 16. *atpB-rbcL* spacer | 804 [777,828] | 909 | 177 (19.5 %) | 52 (5.7 %) | 0.042 | 10 | 75 | 16 |
| 17. *rpl16* intron | 1006 [985,1038] | 1134 | 286 (25.2 %) | 102 (9 %) | 0.058 | 10 | 66 | 16 |
| 18. *rpl20-rps12* spacer | 818 [762,854] | 947 | 221 (23.3 %) | 59 (6.2 %) | 0.047 | 10 | 67 | 16 |
| 19. *accD-psaI* spacer | 818 [491,958] | 1092 | 291 (26.6 %) | 76 (7 %) | 0.062 | 12 | 72 | 16 |
| 20. *ycf3* intron 1 | 738 [730,746] | 781 | 150 (19.2 %) | 46 (5.9 %) | 0.039 | 10 | 72 | 16 |
| 21. *clpP* intron 2 | 637 [618,653] | 710 | 194 (27.3 %) | 55 (7.7 %) | 0.056 | 12 | 77 | 16 |
| 22. *ycf3* intron 2 | 818 [784,871] | 959 | 210 (21.9 %) | 70 (7.3 %) | 0.048 | 10 | 64 | 16 |
| 23. *trnC-petN* spacer | 789 [409,876] | 943 | 277 (29.4 %) | 98 (10.4 %) | 0.07 | 12 | 69 | 16 |
| 24. *atpF-atpH* spacer | 594 [317,652] | 794 | 233 (29.3 %) | 65 (8.2 %) | 0.074 | 12 | 69 | 16 |
| 25. *psaI-ycf4* spacer | 424 [407,449] | 481 | 128 (26.6 %) | 41 (8.5 %) | 0.061 | 10 | 64 | 16 |
| 26. *rbcL-accD* spacer | 626 [502,677] | 769 | 163 (21.2 %) | 44 (5.7 %) | 0.051 | 12 | 69 | 16 |
| 27. *rpoC1* intron | 722 [686,746] | 812 | 167 (20.6 %) | 56 (6.9 %) | 0.044 | 12 | 68 | 16 |
| 28. *trnV* intron | 612 [586,626] | 652 | 130 (19.9 %) | 33 (5.1 %) | 0.039 | 12 | 70 | 16 |
| 29. *psbM-trnD* spacer | 734 [640,781] | 888 | 225 (25.3 %) | 51 (5.7 %) | 0.051 | 14 | 68 | 16 |
| 30. *trnL* intron | 522 [420,544] | 591 | 145 (24.5 %) | 43 (7.3 %) | 0.047 | 12 | 63 | 16 |
| 31. *trnS-trnfM* spacer | 720 [533,790] | 969 | 283 (29.2 %) | 75 (7.7 %) | 0.065 | 14 | 56 | 16 |
| 32. *petB* intron | 764 [739,784] | 832 | 183 (22 %) | 57 (6.9 %) | 0.043 | 14 | 50 | 16 |
| 33. *atpF* intron | 818 [759,854] | 934 | 215 (23 %) | 73 (7.8 %) | 0.052 | 16 | 58 | 16 |
| 34. *psbK-psbI* spacer | 377 [150,405] | 478 | 139 (29.1 %) | 40 (8.4 %) | 0.072 | 12 | 39 | 16 |
| 35. *rpl2* intron | 663 [653,671] | 672 | 27 (4 %) | 6 (0.9 %) | 0.006 | 16 | 35 | 16 |
| 36. *rps12* intron | 540 [540,542] | 543 | 21 (3.9 %) | 4 (0.7 %) | 0.006 | 18 | 35 | 16 |
| 37. *ndhB* intron | 680 [677,683] | 689 | 12 (1.7 %) | 3 (0.4 %) | 0.003 | 18 | 26 | 16 |
| 38. *trnA* intron | 801 [801,801] | 801 | 17 (2.1 %) | 6 (0.7 %) | 0.003 | 20 | 31 | 16 |
| 39. *trnI* intron | 848 [846,852] | 859 | 17 (2 %) | 3 (0.3 %) | 0.003 | 22 | 26 | 16 |
| *rps16* intron | 778 [719,810] | 929 | 189 (20.3 %) | 60 (6.5 %) | 0.052 | NA | 72 | 13 |
| *trnK-rps16* spacer | 431 [332,464] | 538 | 164 (30.5 %) | 43 (8 %) | 0.077 | NA | 56 | 15 |
